# So, It’s Pricier Than Before, but Why? Price Increase Justifications Influence Risky Decision Making and Emotional Response

**DOI:** 10.3389/fpsyg.2019.01883

**Published:** 2019-09-03

**Authors:** Juan C. Salcedo, William Jiménez-Leal

**Affiliations:** Laboratorio de Cognición, Departamento de Psicología, Universidad de los Andes, Bogotá, Colombia

**Keywords:** emotion, decision making, economic bubbles, framing, risk

## Abstract

In this paper, we investigated how justifications for price increases are associated with risky decision making and emotional responses. Across two studies with paired lottery choices and sequential decisions, we found that participants presented with a justification for price increases based on increasing demand decided to invest in a comparatively riskier asset more often than participants presented with a justification for price increases based on increasing tax or those presented with no justification at all. We also found that participants presented with justifications for price increases based on increasing demand also reported higher arousal and displayed higher galvanic skin response than people in the other two justification conditions. Together, these studies provide evidence that only the increasing demand condition underlying a price increase of a risky asset can influence the decision to buy and suggests that emotional activation has a crucial role in such a decision process.

## Introduction

Baijiu, a strong and pungent liquor, is the most popular distilled spirit in China. Suppose you’ve never had baijiu and you happen to read a news article mentioning how baijiu prices have been rapidly increasing in the last couple of months. The next day you are at a bar waiting for some friends and the bartender suggests you try their recently imported baijiu. Would you go ahead and buy a drink of the exotic alcoholic beverage?

Now consider two sets of reasons the article could mention to justify price increases. On the one hand, it could be that prices had been going up because baijiu had suddenly become a popular drink in the domestic market and many people were demanding it. On the other hand, it could be that the prices had been increasing because of recent import tariffs levied on the product in order to protect domestically produced spirits. In which situation do you think you are more likely to buy this drink? When prices go up because of demand or when the price is determined by the taxes set on it? Would you be indifferent to reasons and base your decision only on the price, taste, etc? The reason for the price increase of a product could potentially carry implicit information about the desirability of the product itself, for example through the social reputation of a decision maker (Schöbel et al., 2016). In this paper we examine the extent to which certain reasons for price increases influence a product’s desirability and consequently how these reasons are linked to risky decision making.

We believe differences in price justifications might be responsible for the onset of phenomena like economic bubbles on an individual level. Economic bubbles are evidenced when the underlying assets or goods can be considered investments, that is, when they can be resold. As such, the increasing price of an acquired commercial asset that experiences continually increasing demand implies the possibility of realizing a profit when sold in the market. When an economic bubble expands, the justification for the price increase of the asset in question typically involves a component of increasing demand (Shiller, 2002). This usually triggers a positive emotion in the buyer driven by the prediction of higher gains, which further fuels the growth of the bubble. Is this justification alone generating a significant influence on the decisions that investors make? Specifically, does an explicit reason for the increase in the price of an asset, regardless of its soundness, affect risk aversion, and does the message conveyed in that reason influence decision making under risk?

During a bubble expansion, more people invest in the underlying asset and everyone has to invest comparatively larger amounts of money as the price of the asset increases. Assuming constant probabilities and risk parameters, such behavior entails a corresponding decrease in risk aversion as stake size increases. This stands in contrast to the evidence regarding greater relative risk aversion with higher stake sizes (Binswanger, 1981; Kachelmeier and Shehata, 1992; Holt and Laury, 2002) and seems to contradict the predictions of prospect theory in the gains domain (Kahneman and Tversky, 1979; Tversky and Kahneman, 1981). Many causes of the “irrational” decision to invest ever larger quantities of money in an expanding bubble have been proposed, including the effect of positive valence emotions (Akerlof and Shiller, 2010), the link between emotional arousal and risky decision making (Bechara et al., 1997; FeldmanHall et al., 2016), the relationship between emotional states and risky decision making (Schlösser et al., 2013; Stanton et al., 2014), as well as social herding models (Baddeley, 2010).

On the one hand, positive valence emotions have been hypothesized to promote peripheral information processing, leading individuals to rely more heavily on factors such as the source of the information compared to its content when making a decision (Petty and Cacioppo, 1986). Furthermore, it has been shown that inducing positive emotions leads to greater risky decision on gambling tasks in the lab (Yuen and Lee, 2003; Chou et al., 2007). Thus, it could be argued that betting that an asset, which has already seen an increase in its price, will continue to see its value grow could potentially result in arousal and positive valence emotions that lead investors to comparatively risky decision making. It has also been proposed that emotional arousal is linked to risky decision making (Loewenstein et al., 2001) with empirical evidence to support the notion that greater emotional arousal is tied to increased risky decision making in the lab (Figner et al., 2009). On the other hand, herd behavior has been identified as a major contributor of investment decision making in commodities markets, crowdfunding platforms, information security markets and stock markets (e.g., Demirer et al., 2015; Lei et al., 2017; Papapostolou et al., 2017; Vo and Phan, 2017; Shao et al., 2019). Herd behavior models suggest that people follow others and imitate group behavior rather than make individual investment decisions based on their own private information.

Within financial markets, such herd behavior generates instability and episodes of speculation that can transform into bubbles (Baddeley, 2010). If the reason for a price increase carries implicit information of the behavior of others, it could potentially trigger herd behavior. Moreover, it has been hypothesized that the underlying mechanism driving herd behavior is emotional in nature. More specifically, emotional contagion (Hatfield et al., 1993), has been postulated as a possible trigger of herd behavior of various social phenomena including economic bubbles (Kameda et al., 2015), and Baddeley (2010) has argued that herding in humans can be conceptualized as a proximate mechanism motivated and precipitated by emotional responses. Furthermore, within a neurobiological framework of value-based decision making, herd behavior may constitute a habit valuation system than competes with goal-directed valuation systems aimed at maximizing profit (Rangel et al., 2008) as in all real-world decisions. Additionally, the inherent risk and uncertainty involved in economic bubble contexts act as modulators of the habit and goal-directed valuation systems that ultimately play a vital role in financial decision making (Rangel et al., 2008).

However, the effect of the justification for the price increase has not been considered as a major determinant of risk preferences in an environment with progressively increasing prices (as in expanding bubbles or inflationary periods). Such a justification would act as a framing effect (Tversky and Kahneman, 1981). Moreover, even though the effect of emotions on economic behavior has been analyzed via physiological measurements (Crone et al., 2004; Smith and Dickhaut, 2005), the relationship between price increase justifications and emotions has not been investigated and research on how the reason for a price increase could affect economic decisions and influence emotional responses has not been conducted in an experimental setting. This issue has several economic applications; its implications for decision making during financial bubbles are discussed here.

This paper studies behavior in paired lottery choice experiments to test whether specific price increase justifications alter individuals’ financial decisions under risk. Specifically, we are interested in whether changes in the justification for the increase in prices during a series of sequential decisions affect risk aversion and emotional response. We hypothesize that people who are told that prices are increasing because people are buying more of a given asset will take more risks than those who read that prices are increasing due to an alternative reason or are not given a reason for the price increase (Hypothesis 1), and people who read that prices are increasing because people are buying more of a given asset will have a greater emotional response than those who read that prices are increasing due to an alternative reason or are not given a reason for the price increase (Hypothesis 2). Raw data for both experiments is included in the Supplementary Tables. All materials, data sets and codes for analyses are available in the Open Science Framework Repository^1^.

This study was carried out in accordance with the recommendations of the ethical committee of the Faculty of Social Sciences of the Universidad de los Andes, with written informed consent from all subjects. All subjects gave written informed consent in accordance with the Declaration of Helsinki.

## Experiment 1

### Materials and Methods

A total of 162 students from several universities in Bogotá (Colombia) were recruited primarily through informal announcements such as Facebook, word of mouth and email. Mean age of participants was 23.2 (*SD* = 4.96) and 58% were female.

This study was conducted online over a period of 3 weeks using a Qualtrics interface. In the instructions, participants read that they would have to make a series of economic decisions over 10 rounds. They were also informed that one of the participants would be randomly selected and that this person would be paid the amount of money from one of their 10 rounds (also selected at random) by the researchers conducting the experiment (this same payment protocol was followed in Experiments 1 and 2). This setup was based on the paired lottery choice decisions task first designed by Holt and Laury (2002) to yield a measure of their risk preferences. Although the task was inspired by the original Holt and Laury risk preference task, the expected value of the products over the 10 rounds does not follow the same tendency observed in the original task (where the expected value of the risky option eventually surpassed that of the safe option) because the goal of this study was to isolate the effect of the price increase justification on risky decision making. Therefore, in this case, the task involved a decision between investing in product W which offered a lesser amount of money with complete certainty (100% probability) and investing in product X, which offered a substantially larger amount of money with less certainty (20% probability). At every round, the decision involved greater amounts of money than in the previous round for both products, such that the amounts at stake increased sequentially over the 10 rounds even though the probabilities remained constant (the payment matrix increased sequentially in an exponential manner to resemble price increase behavior in economic bubbles). Crucially, the safe option (product W) always offered a greater expected value that the risky option (product X) in each of the 10 rounds. Additionally, the amounts in question were considerable; by round 10 the potential gain of product X equaled almost half the minimum monthly wage in Colombia (which at the time of the study was about USD 220) and the sure gain of product W equaled about three times the minimum daily wage in Colombia (which at the time of the study was about USD 20). In all three conditions, participants received no feedback regarding the outcome of their investment decisions across all 10 rounds. Outcome information was purposefully withheld in order to avoid the potential influence of a win or a loss in any given round on posterior decision making. This allowed us to isolate the effect of the justification for the price increase on decision making.

Participants were randomly assigned to one of three groups; two treatment conditions and a control condition. Each of the treatment conditions consisted of a different justification regarding the reason for the price increase of product X as participants moved from round 1 to round 10. In the control condition no justification was provided. In all three conditions the question in round 1 was the same: “In which of these two products do you prefer to invest?” Likewise, in the instructions for all three conditions it was never stated that the participants would be playing with or against each other. Furthermore, the justifications between rounds in all three conditions included no explicit statements that could lead participants to believe that the information provided corresponded to other participant’s behavior.

The first condition was the “Increasing Demand Justification” group. Participants assigned to this treatment (*N* = 52) were instructed to complete the series of 10 paired lottery choice decisions, but between round 1 and round 2 they read the following: “More people are buying product X. The demand for product X has increased, therefore, the prices for product X have increased for round 2.” Between all other rounds, they read the following: “Even more people are buying product X. The demand for product X keeps increasing, therefore, the prices have increased for the next round.” The purpose of this between-rounds frame was to provide a reason for the price increase based on the fact that more people buying the product was increasingly pushing demand upward and hence generating a price hike.

Participants assigned to the second condition (*N* = 53), “Increasing Tax Justification,” completed the exact same paired lottery choices over the same 10 rounds with the same probability and amount parameters. However, instead of the increasing demand message between rounds, they read the following between round 1 and round 2: “The government has decided to impose a tax on product X. The cost of product X has risen, and the prices have therefore increased for round 2.” Between all other rounds, they read the following: “The government has increased the tax on product X. The cost of product X keeps increasing, therefore, the prices have increased for the next round.” The purpose of this between-rounds frame was to provide an alternative justification for a price increase that could be plausible but that, at the same time, did not involve an explicit rise in demand (as in the first treatment group) nor an implicit rise in demand (such as might be inferred by participants in the case of an inflation or a supply reduction).

Finally, in the control condition (“No Justification”), participants (*N* = 57) completed the lottery choice questions over the 10 rounds but were given neither the Increasing Demand nor Increasing Tax justifications between rounds. Nevertheless, exactly like in the other two conditions, the sequential increase in price over the 10 rounds was made explicit.

Once participants finished the 10 rounds, they were presented with a series of eight statements, randomly presented, and they had to report how much they agreed with each statement on a Likert scale within the same Qualtrics interface. These eight items constituted a self-report of emotional response experienced during the decisions they had just made. These statements were constructed based on the Risk as Feeling hypothesis (Loewenstein et al., 2001), which posits that emotional reactions to situations that involve risk tend to diverge from the cognitive assessments of those risks and that, in such cases, emotional responses often drive behavior. The purpose of these items was to probe for self-reported emotional response at the time of decision making. They included statements explicitly associated with emotional states, but also with risky situations associated with positive emotions (Schonberg et al., 2011). The eight items closely followed the statements utilized by Figner et al. (2009) to assess self-reported decision strategies and self-reported decision-related emotional response in risky decision making. Specifically, participants answered how much they agreed with the following statements: “I made the decisions intuitively,” “I made the decisions mathematically” (reverse coded), “I felt enthusiasm making the decisions,” “I felt euphoria making the decisions,” “The feeling while making the decisions was similar to driving a car at high speed,” “The feeling while making the decisions was similar to gambling in a casino,” “Decision making was a pleasant experience,” “Decision making was difficult.”

### Results

To measure the degree of risk aversion we observed how many times each of the participants chose the risky product (product X) instead of the safe product (product W) over the 10 rounds. Each of these was considered a risky decision. Then the number of risky decisions made by all participants in all three groups was directly compared.

**FIGURE 1:**
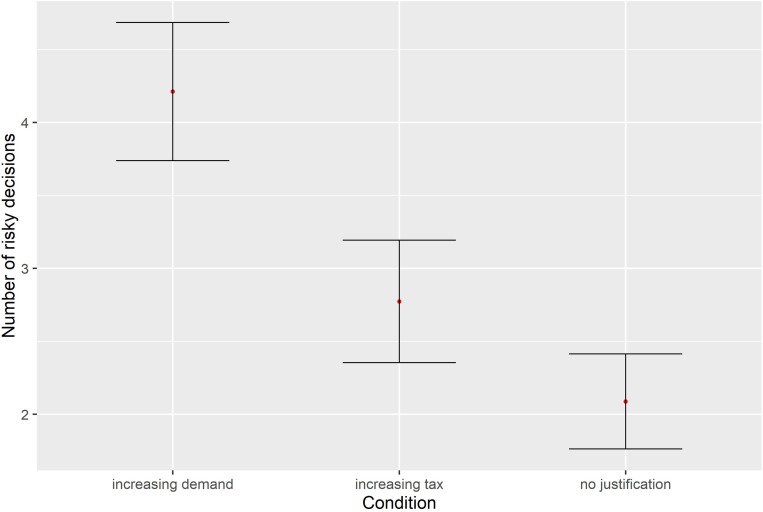
Mean number of risky decisions per condition. Error bars represent standard errors.

In Table 1 and Figure 1 we report the number of risky decisions (out of 10 possible decisions) that participants made under each of the treatment frames and the control group. Levene’s test confirmed a slight violation of the homogeneity of variance assumption [*F*(2,159) = 3.05, *p* = 0.05] and we therefore report the Brown-Forsythe statistic for the variance analysis and performed *post hoc* comparisons.

**TABLE 1:** Means, medians and standard deviations, for risky decisions.

**Condition**	**Mean**	**Median**	***N***	***SD***
Increasing demand	4.21	4	52	3.41
Increasing tax	2.77	1	53	3.05
No justification	2.09	1	57	2.46

**SD* indicates standard deviation.*

The corresponding 3 (Condition: Increasing Demand, Increasing Tax and No Justification) X 2 (risky and not risky) ANOVA, estimated with the Brown Forsythe Correction, indicated a main effect of frame over the number of risky decisions *F*(2,145.10) = 6.98, *p* = 0.001, η^2^ = 0.08. A *post hoc* contrast test (Bonferroni correction) indicated that adding a justification (Increasing Demand or Increasing Tax) significantly increased the number of risky decisions that participants made *t*(144.68) = 3.05, *p* = 0.001, *d* = 0.46. The Increasing Demand Justification significantly increased the number of risky decisions vs. the No Justification condition *t*(91.92) = 3.69, *p* < 0.01, *d* = 0.61, but there was no significant difference between the Increasing Tax and No justification conditions *t*(91.92) = 1.3, *p* = 0.19, *d* = 0.28. Crucially though, the Increasing Demand Justification frame increased the number of risky decisions compared with the Increasing Tax Justification *t*(101.29) = 2.27, *p* = 0.02, *d* = 0.43. Additionally, the linear tendency, which indicates that the frame affects the number of risky decisions is both significant and in the expected direction *F*(1,159) = 7.01, *p* < 0.001. A sensitivity analysis was run [with the software G^∗^Power (Faul et al., 2007)] to identify the boundary conditions of our inferences. More precisely, we ran a sensitivity analysis which allows the user to determine the plausible effect size given an alpha level, sample size and desired power. This analysis revealed that assuming a power of 95%, with an alpha level of 5% and our sample size, our study has a minimum detectable effect of *f* = 0.3. We also computed the ν statistic as a way to estimate the accuracy of our estimates (Davis-Stober and Dana, 2014; Lakens and Evers, 2014) independently of the observed significant result. The ν statistic has also been suggested as an indication of the replicability of a finding (Davis-Stober and Dana, 2014), when considered as a measure of the stability of an estimator. Given that we estimated three parameters with the ANOVA model, with an observed *r*^2^ of 0.08, this results in a ν = 0.73. This statistic varies in a range from 0 to 1, where 1 indicates a high stability of the model parameters and an increased chance of replication.

**FIGURE 2:**
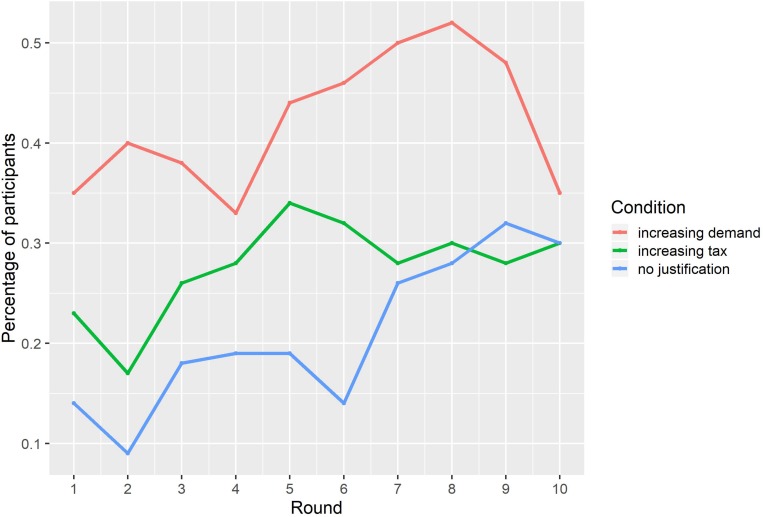
Percentage of participants who selected the risky option in each round.

In addition, we found a clear difference in the tendency to choose the risky product over the 10 rounds (Figure 2). In particular, in each of the 10 rounds, the absolute number of participants who selected the risky option in the Increasing Demand Justification frame was greater than in the Increasing Tax Justification and in the No Justification frames. Likewise, in the Increasing Demand Justification frame, the percentage of participants who selected the risky product increased constantly over 4 continuous rounds (between round 4 and round 8), one more round than in any of the bullish runs exhibited by participants in the other two frames.

To appropriately capture the dynamic nature of the decision-making process in this situation, we fitted a lagged dependent variable regression model so as to account for prior values of the dependent variable (e.g., choice in the previous round).^2^ This model allows us to examine the predictive value of the condition, conditional on given knowledge of the previous choice (Finkel, 1995). As expected, prior choice was a good predictor of subsequent choices (β = 0.47, *p* < 0.05) and there was a significant difference between the increasing demand frame and the control condition (β = 0.46, *p* < 0.05) but not between the tax frame and the control condition (β = 0.11, *p* = 0.41). Given that participants only received information regarding the Justification for the price increase after round 1, the decisions participants made in the first round could not have been affected by the justifications (or lack thereof) for price increases. Therefore, we also fitted the model without the information from round 1 (i.e., taking into account only the decisions participants made in rounds 2–10) and, in this case, differences between experimental conditions and control conditions persist in the presence of the lagged variable.

**FIGURE 3:**
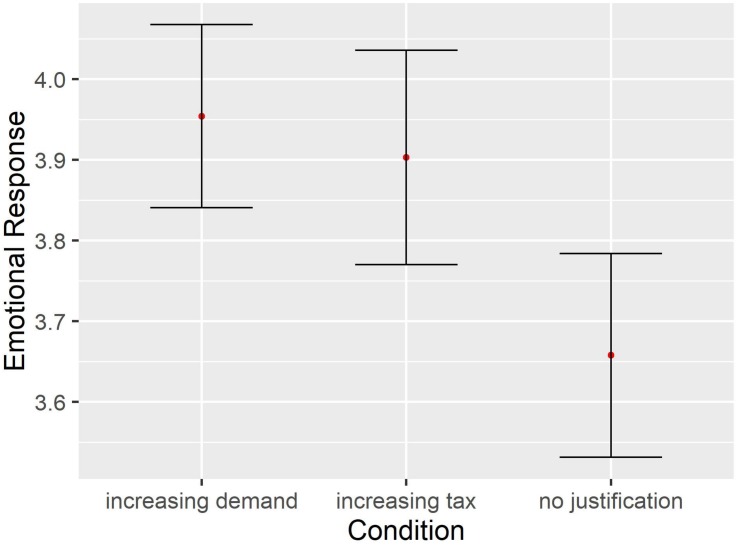
Self-report of emotional response. Error bars represent standard errors.

The self-report emotion questions were analyzed in conjunction and an indicator of emotional response was constructed based on an average of the 8 questions (Cronbach’s α = 0.66, including our reverse coded item). As can be observed in Figure 3, the emotional response indicator reflected a tendency in the expected direction (i.e., greater emotional response for those participants who made the decisions under the Increasing Demand Justification group compared to other two frames) but the corresponding analysis of variance was not significant *F*(2,159) = 1.69, *p* = 0.19, η^2^ = 0.02. We also performed an exploratory factor analysis on the self-report questions, to verify the assumption of a single underlying latent factor. This analysis suggested the presence of two factors, where one of them seems to tap more directly into emotional arousal and comprises only three of the eight questions (*enthusiasm*, *euphoria* and *feeling driving a fast car*, Cronbach’s α = 0.73). A one way ANOVA with this alternative indicator of emotional arousal is consistent with the previous analysis and fails to identify an effect of the Condition [*F*(2,159) = 2.41, *p* = 0.09, η^2^ = 0.03], while exhibiting the same pattern of differences presented in Figure 3. Results with the average of the questions of the second factor, seemingly related to emotional valence, do not follow a similar pattern and result in a very different outcome when submitted to an ANOVA (*F* < 1).

However, we did find an important difference in the percentage of participants that made the decisions in both justification frames who reported increased emotional arousal (Increasing Demand = 54%, Increasing Tax = 51%), compared with the percentage of participants that made the decisions in the No Justification frame who reported increased emotional arousal (33%). Similarly, the percentage of participants that made the decisions in both justification frames reported feeling more of a pleasurable emotion (Increasing Demand = 40%, Increasing Tax = 45%) compared with the percentage of participants that made the decisions in the No Justification frame (32%). These differences do not reach statistical significance (χ*2* < 1) but constitute a clear pattern suggesting a connection between risk and emotion.

### Discussion

The results of experiment 1 suggest that information regarding the quantity of people buying a given asset seems to drive risky decision making more than information regarding taxation of that same asset. We observe this effect while keeping constant the probabilities and the expected value associated with the decisions. Moreover, given the results of study 1, it is reasonable to assume that people are also sensitive to the information itself, even limited impoverished information such as the one we used in this study. The information (the justification) seems to influence people’s decisions to invest in a particular asset beyond the price and the perceived utility. As can be evidenced in Figure 2, there was a drop in risky decision making in the final two rounds for the Increasing Demand condition. Given that participants knew *a priori* that the task would run for a total of 10 rounds, it could be argued that this drop reflects an effort to compensate for the elevated risky decision making in earlier rounds compared to the other two conditions. Nevertheless, there is an observed risk seeking tendency in the three conditions as participants progressed through the 10 rounds (not including the last rounds of the treatment condition mentioned above). This tendency could be explained by an anticipated regret (Zeelenberg, 1999) for missing out on a large gain (i.e., from not choosing the risky option), especially given the fact that they received no sequential outcome feedback and would potentially realize the gains of only one out of their 10 choices. This characteristic of our experimental paradigm does not allow us to model how sequential time periods influence relative risk aversion as they would in a real-world economic bubble, but it does allow us to examine how the justifications for the price increases affect risk aversion.

In experiment 1, emotional response was measured with a set of questions aimed at measuring both emotional arousal and emotional response. Failure to detect differences in emotional response could have been caused by the inherent ambiguity of these questions. Namely, it is possible that the questions failed to describe the quality and quantity of the emotional response participants experienced. In order to tackle this issue in experiment 2, we used galvanic skin conductance as a proxy of emotional arousal (Figner and Murphy, 2010; Starcke et al., 2010) and the Self-Assessment Manikin (Bradley and Lang, 1994) for emotional valence.

## Experiment 2

### Materials and Methods

A total of 39 undergraduate students from a large private university in Colombia were recruited via word of mouth. Mean age of participants was 21.79 (*SD* = 3.03) and 59% were female. Sample size was determined based on the availability of lab resources and it is comparable with studies using similar techniques (Bechara et al., 1997; Krosch et al., 2012).

As in Study 1, participants were randomly assigned to each of the three same frame groups of the first study (Increasing Demand Justification, Increasing Tax Justification and No Justification). All participants were fully informed of the details of the procedure, which took place in the lab, and received a chocolate bar after signing a consent form as compensation for participating. Moreover, as in the first study, participants were informed that one of the participants from study 2 would be randomly selected and that this person would be paid the amount of money from one of their 10 rounds (also selected at random) by the researchers conducting the experiment. All participants made the same series of 10 decisions in a computer in the lab while connected to two electrodes on their non-dominant hand. We decided to use Galvanic Skin Response (GSR) as a proxy of emotional arousal. GSR is used to study affective processes because the autonomic nervous system plays a significant role in emotion and motivation. Changes in electro-dermal activity and skin conductance are related to changes in eccrine sweating, which are in turn related to activity in the sympathetic branch of the autonomic nervous system. Accordingly, GSR measures have been used to study psychological processes related to sympathetic arousal in general (Figner and Murphy, 2010), and in tasks evaluating risky decision making in particular (Jenkinson et al., 2008; Figner et al., 2009).

We used the Biolink 1100 hardware and AcqKnowledge 1100 software to analyze galvanic skin conductance variations of participants. In order to establish a baseline skin conductance reading, all participants were first presented with the same relaxing landscape video for a period of 3 min and 30 s. Following the video, participants read the experiment’s instructions and proceeded to make their decisions over the 10 rounds. Afterward, all participants selected a visual self-report query whose aim was to establish emotional valence. This query was constructed with Qualtrics based on the Self-Assessment Manikin first elaborated by Bradley and Lang (1994), which has been previously used in a study of a risk aversion in finance professionals (Cohn et al., 2015). Specifically, this question asked them to indicate their emotional state in a schematic representation of a face. The data obtained with this query was numerically coded in a scale from −2 (corresponding to the saddest looking face), −1, 0, 1, and 2 (corresponding to the happiest looking face).

### Results

Decision data of this study follows the same trend of our findings in experiment 1. Participants who made the decisions in the Increasing Demand Justification condition made more risky decisions [*M* = 5.31, 95% *CI* (3.54,7.08)] than the participants who made the decisions with the Increasing Tax Justification condition [*M* = 2.77, 95% *CI* (1.78,3.76)] and more than participants who made the decisions with No Justification [*M* = 4.0, 95% *CI* (2.98, 5.02)]. The corresponding variance analysis indicated a main effect of frame over the number of risky decisions *F*(2,36) = 4.46, *p* < 0.05, η^2^ = 0.20. However, a *post hoc* test only indicated a significant difference between the number of risky decisions made by participants in the Increasing Demand and Increasing Tax conditions but found no significant difference between the number of risky decisions of the Increasing Demand and the No Justification conditions. The corresponding visual self-report data of the subsequent question also seems to indicate a marginally greater positive valence emotion for those participants who made the decisions with the Increasing Demand Justification [*M* = 0.85, 95% *CI* (0.36, 1.33)] than for those participants who made the decisions with the Increasing Tax Justification [*M* = 0.69, 95% *CI* (0.12, 1.26)] and for those participants who made the decisions with No Justification [*M* = 0.69, 95% *CI* (0.24, 1.15)]. Likewise, these differences were not statistically analyzed given the reduced number of participants per condition (*N* = 13).

For the physiological analysis, the pre-established protocol package of the AcqKnowledge software was utilized in order to calculate dermo-galvanic skin response from the skin conductance raw data obtained with the electrodes. The magnitude of the dermo-galvanic skin conductance was determined by measuring the area under the skin conductance curve (Figner and Murphy, 2010). Every skin conductance session was divided in two phases: a baseline (established with the relaxing video previously mentioned) with a total duration of 210 s and a decision-making phase with an average duration of 200 s. In Table 2 we report the area under the curve (μS/second) for each of the three frames in both phases.

**TABLE 2:** Dermo-galvanic skin response.

**Frame**	**Baseline**	***SD***	**Task**	***SD***	***N***
Increasing Demand	154.06	236.41	1083.59	562.67	13
Increasing Tax	272.82	266.01	728.03	465.77	13
No Justification	242.63	290.12	660.48	602.72	13

**SD* indicates standard deviation.*

**FIGURE 4:**
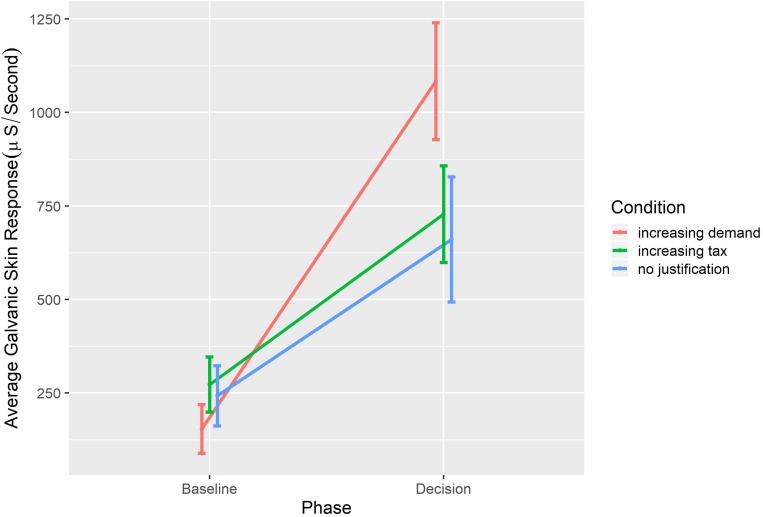
Interaction effect of dermo-galvanic response in both phases for each of the three frames.

Two mixed effects models (Bates et al., 2015) were fitted to analyze these data with phase (baseline, task) as a within-participants factor, condition (Increasing Demand, Increasing Tax, No Justification) as a between participants factor, and Dermo-Galvanic Skin Response (area under the curve standardized per second) as a dependent variable. The difference between the models was the inclusion of an interaction term between phase and condition. The inclusion of the interaction term resulted in a significant change [χ^2^(2) = 11.65, *p* < 0.001] and better fit (*AIC* = 1173 and 1165.3, respectively). The fixed coefficient for the interaction between Condition and Phase is significant [*F*(2,36) = 6.27, *p* < 0.001]. This last interaction effect can be visualized in Figure 4 and clearly indicates that the Increasing Demand frame resulted in a higher arousal compared to the other conditions.

Independent ANOVA tests indicated no significant difference in the dermo-galvanic skin response during the baseline phase *F*(2,36) = 0.71, *p* = 0.50, η^2^ = 0.04, nor during the decision phase *F*(2,36) = 2.25, *p* = 0.12, η^2^ = 0.11. However, a paired *t*-test revealed a significant difference in the dermo-galvanic skin response between the baseline and the decisions across conditions *t*(38) = −8.08, *p* < 0.001, *d* = 1.29.^3^ An analysis of variance performed on the galvanic response in the decision phase identified significant differences. Pairwise comparisons indicated that the increase in dermo-galvanic skin response for those participants who made the decisions with the Increasing Demand Justification (*M* = 929.53, *SD* = 477.17) was significantly larger than the increase in dermo-galvanic skin response exhibited by those participants who made the decisions in the Tax Increase Justification (*M* = 455.21, *SD* = 383.47) and larger than that exhibited by the participants who made the decisions with No Justification (*M* = 417.85, *SD* = 362.56), which can be visualized in Figure 4.

We fitted a linear model in order to predict the number of risky decisions based on the increase in dermo-galvanic skin response and condition. This model resulted in a significant model [*F*(3,75) = 5.33, *p* < 0.001, *r*^2^ = 27%] with a significant effect of the dermo-galvanic skin response (*t* = 2.91, *p* < 0.01). The number of risky decisions increased in 0.003 with every additional μS/second of dermo-galvanic skin response. There was no effect of condition (increasing demand, *t* = −1.3, *p* = 0.21; *t* = 0.9, *p* = 0.37), but there was an interaction between dermo-galvanic response and condition (see Table 3).

**TABLE 3:** Mixed effects model for SCR.

**Predictor**	**Estimates**	**CI**	***p***
(Intercept)	242.63	9.07–476.19	0.042^∗^
Increasing Demand	−88.56	−418–241.74	0.599
Increasing Tax	30.19	−300.12–360.49	0.858
Decision	417.85	194–641.14	< 0.001^*⁣**^
Condition Increasing Demand: Decision Phase	511.67	195.89–827.46	0.001^∗∗^
Condition Increasing Tax: Decision Phase	37.36	−278.43–353.14	0.817
**Random Effects**			
σ^2^	84375.45		
τ_00 Condition:participant_ID_	17852.10		
τ_00 participant_ID_	82388.69		
ICC _Condition:participant_ID_	0.10		
ICC _participant_ID_	0.45		
Observations	78		
Marginal R^2^/Conditional R^2^	0.374/0.714		

*^∗^*p* < 0.05; ^∗∗^*p* < 0.01; ^∗∗∗^*p* < 0.001.*

**FIGURE 5:**
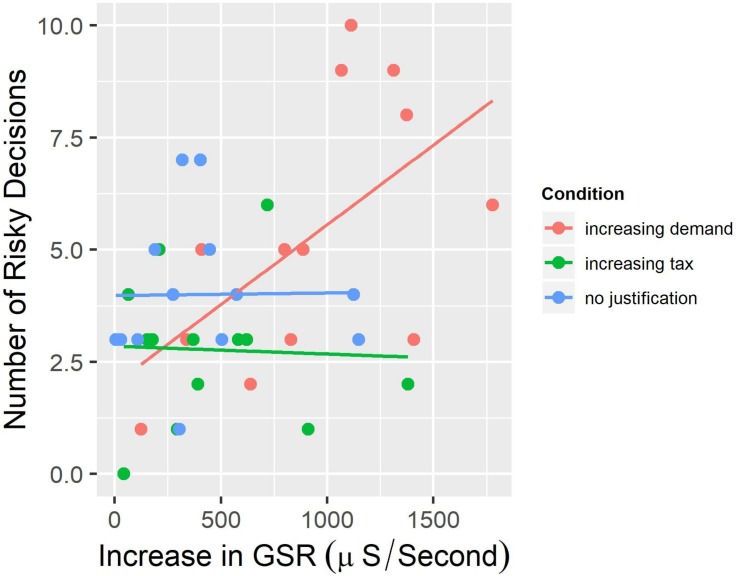
Relationship between the increase in dermo-galvanic skin response and the number of risky decisions by condition.

We did not find a significant relationship between the increase in dermo-galvanic skin response and the number of risky decisions for subject in the Increasing Tax Justification group (*r* = −0.04, *p* = 0.90), nor for the participants in the No Justification group (*r* = −0.01, *p* = 0.97) (see Figure 5).

In order to assess the consistency between reported emotions and physiological response, we analyzed the correlations between self-reported emotions (more precisely, the emotional valence measured via the visual self-report query, labeled as valence from now on) and the general measure of emotional arousal (more precisely, the activation difference in SCR between test and baseline) per condition. This analysis shows that there is a significant strong correlation for the increasing tax condition, but not for the increasing demand condition (the same pattern occurs when considering only the test condition, see Table 4). This suggests that as the tax increases, people experience more negative affect, preventing them from making investing decisions. Thus, emotion arousal fulfills a signaling role in our experimental setting. A linear model with self-reported emotion as a dependent variable, and condition and increase in arousal difference as predictors reveals only a significant interaction between condition and phase. That is, while mean valence does not differ between conditions or as a function of general measure of activation only, for the tax condition the expected mean is lower than for the other conditions only in the presence of the arousal difference *F*(2,330) = 4.70, *p* < 0.05, η^2^ = 0.19.

**TABLE 4:** Correlations between self-reported emotions and valence.

**Frame**	**1**	**2**	**3**
(1) Increasing Demand	0.0771		
(2) Increasing Tax		−0.595^∗^	
(3) No Justification			0.395

*Valence = activation difference in SCR between test and baseline. ^∗^*p* < 0.01.*

## General Discussion

The results of these two studies indicate that individuals who read that prices are increasing because people are buying more of a given asset will take more risks than those who read that prices are increasing due to an alternative reason or are not given a reason for the price increase (Hypothesis 1) and individuals who read that prices are increasing because people are buying more of a given asset will have a greater emotional response than those who read that prices are increasing due to an alternative reason or are not given a reason for the price increase (Hypothesis 2).

In study 1, we found that the average number of participants and the average number of risky decisions that these participants made were both considerably higher in the Increasing Demand Justification condition compared with the other two conditions. The fact that the Increasing Demand Justification constitutes, in essence, a narration of herd behavior provides support for the idea that this justification might work as a sort of social influence signaling mechanism that generates a feedback loop in which people become progressively less risk averse. This effect might be even stronger in real economic bubbles where investors can corroborate the veracity of an increasing demand. Herd behavior engenders behavior imitation. Such imitation allows learning of particular behaviors in a quicker and more effective manner than comparable individual learning mechanisms in social environments (Baddeley, 2010). Herd behavior might also work as an instrument for fast information broadcasting concerning the localization and availability of resources (Danchin et al., 2004; Surowiecki, 2004) and even allows for social cohesion, which Simon (1990) considered an evolutionary advantage. In this sense, receptivity to social influence would allow a group of individuals to behave in an altruistic manner, promoting empathy while avoiding selfish behavior. Hence, behavior would therefore contribute to the resolution of social conflicts and help to overcome environmental obstacles.

In study 2, we confirmed that the Increased Demand Justification elicited greater emotional arousal from individuals, inferred from the corresponding galvanic skin response. We also found a correlation between the number of risky decisions and the emotional response. This might indicate that the decision process has a crucial emotional component. Nevertheless, it is impossible to confirm whether the emotional response was triggered by the decision or whether the emotion preceded and (in part or in whole) determined the decision. Regardless, the magnitude of the emotional response is directly related to the justification for the price increase. The crucial role that emotions play in decision making in cases of herd behavior might be better explained as a fast and frugal heuristic (Gigerenzer and Goldstein, 1996), which provides adaptive benefits in various forms. In this case, emotions would be playing a fundamental role in the preservation of herd behavior and would therefore be valuable from an evolutionary point of view. As Frank (1988) suggests, emotions would be acting as strategic commitment devices forcing individuals to act in an apparently irrational way, but ultimately fulfilling a very important adaptive function. This would be in line with theories of risky decision making driven by emotional responses (Loewenstein et al., 2001), emotional contagion precipitating herd behavior (Kameda et al., 2015), and emotions potentiating risky decision making via herd behavior (Baddeley, 2010).

A vital component of these two studies was that participants never knew whether they had won or lost their money in a given round. In this sense, these two studies manage to discern between the justification framing effect and the potential effect caused by winning and/or losing. Irrespective of their own decisions, the prices of both products kept rising over the course of the ten rounds and participants gained no information whatsoever concerning the results of their decisions. Hence, we can infer that the justification for the price increase alone managed to influence the decisions of the participants.

The next steps to validate our finding include the use of more fine-grained emotion measures. Valence and arousal are only two of the dimensions of an emotion, and recent literature in decision making has highlighted the need to include discrete emotion models in the explanation of decision making (Lerner et al., 2015). Similarly, emotion inductions can shed light on the causal direction of the association detected between price justification and arousal. Likewise, as a complement to the study of positive valence emotions, future research would greatly benefit by separately analyzing the correlation with or influence of distinct negative valence emotions on risky decision making.

There are four main limitations of this study that should be considered in interpreting these results. First, small sample sizes limit the possibility of interpretation of effect sizes. This study should not be read as a confirmatory but as an exploratory study and as such, it is contributing to generating a hypothesis, not as its litmus test. Second, the nature of emotion we have assumed must be considered. With our measures we assume a dimensional nature of emotion (Russell and Barrett, 1999), thus focusing on arousal in experiment 1 and on arousal and valence in experiment 2. These should be more carefully studied and dissociated in future research. Third, our main manipulation. Even though the tax and demand manipulations are equivalent in both their payoff matrices and the linear increases associated, it is possible that participants considered the demand condition to be true within the terms of the experiment but not the tax condition. If this was the case, risky decisions and emotional activation variation would have been a function of the assumed realism of one of the conditions, not of the bubble effect ***per se***. However, in this situation, the differences between the tax and control conditions would remain unexplained. This alternative interpretation highlights, however, that the mechanism that might underlie herd behavior in bubble situations has to do with the perception of a relevant group of reference against which one’s risky behavior appears normal. Finally, even though our findings are in line with value-based frameworks of decision making, whereby emotional responses precipitate herd behavior and drive risky decision making, our study is not able to discern a causal relationship between emotional response and risky decision making or herd behavior. Furthermore, recent research has highlighted the importance of task relevance on the relationship between emotional valence and behavior (Mirabella, 2018; Wispinski et al., 2018). Hence, future studies should uncover how emotional valence impacts decision making within sequential monetary choice paradigms, on the one hand, and understand the direction and nature of the relationship between emotion, herd behavior and risky decision making on the other.

## Ethics Statement

This study was carried out in accordance with the recommendations of the ethical committee of the Faculty of Social Sciences of the Universidad de los Andes, with written informed consent from all subjects. All subjects gave written informed consent in accordance with the Declaration of Helsinki. IRB Committee: Comité de Ética de la Facultad de Ciencias Sociales de la Universidad de los Andes. The experiments were approved by the Committee in their session of September 2015. All participants signed and informed consent provided either electronically (experiment 1) or in paper (experiment 2).

## Author Contributions

JS proposed the hypothesis, conducted the experiments, and drafted the manuscript. Both authors designed the experiments, contributed to data analysis, and approved the final version of the manuscript for submission. WJ-L provided the critical revisions.

## Conflict of Interest Statement

The authors declare that the research was conducted in the absence of any commercial or financial relationships that could be construed as a potential conflict of interest.

## Footnotes

1 https://osf.io/uexh5/

2 We are very grateful to the reviewers for this suggestion.

3 Notice that this effect size would imply a statistical power of 99% to detect the effect, which is unlikely and suggests the effect size is inflated (Hedges correction for bias gives the same effect size value). Given the exploratory nature of this study, we do not consider it an observation of the true effect, but an estimation of the existence of this effect in a setting with a possibly large sampling error.

## Supplementary Material

TABLE S1 Raw data experiment 1.

TABLE S2 Raw data experiment 2.
